# Non-invasive Brain Stimulation for Central Neuropathic Pain

**DOI:** 10.3389/fnmol.2022.879909

**Published:** 2022-05-19

**Authors:** Qi-Hao Yang, Yong-Hui Zhang, Shu-Hao Du, Yu-Chen Wang, Yu Fang, Xue-Qiang Wang

**Affiliations:** ^1^Department of Sport Rehabilitation, Shanghai University of Sport, Shanghai, China; ^2^School of Mechanical and Automotive Engineering, Shanghai University of Engineering Science, Shanghai, China; ^3^Department of Rehabilitation Medicine, Shanghai Shangti Orthopaedic Hospital, Shanghai, China

**Keywords:** rTMS, tDCS, central neuropathic pain, analgesic mechanism, analgesic effects

## Abstract

The research and clinical application of the noninvasive brain stimulation (NIBS) technique in the treatment of neuropathic pain (NP) are increasing. In this review article, we outline the effectiveness and limitations of the NIBS approach in treating common central neuropathic pain (CNP). This article summarizes the research progress of NIBS in the treatment of different CNPs and describes the effects and mechanisms of these methods on different CNPs. Repetitive transcranial magnetic stimulation (rTMS) analgesic research has been relatively mature and applied to a variety of CNP treatments. But the optimal stimulation targets, stimulation intensity, and stimulation time of transcranial direct current stimulation (tDCS) for each type of CNP are still difficult to identify. The analgesic mechanism of rTMS is similar to that of tDCS, both of which change cortical excitability and synaptic plasticity, regulate the release of related neurotransmitters and affect the structural and functional connections of brain regions associated with pain processing and regulation. Some deficiencies are found in current NIBS relevant studies, such as small sample size, difficulty to avoid placebo effect, and insufficient research on analgesia mechanism. Future research should gradually carry out large-scale, multicenter studies to test the stability and reliability of the analgesic effects of NIBS.

## Introduction

**FIGURE 1:**
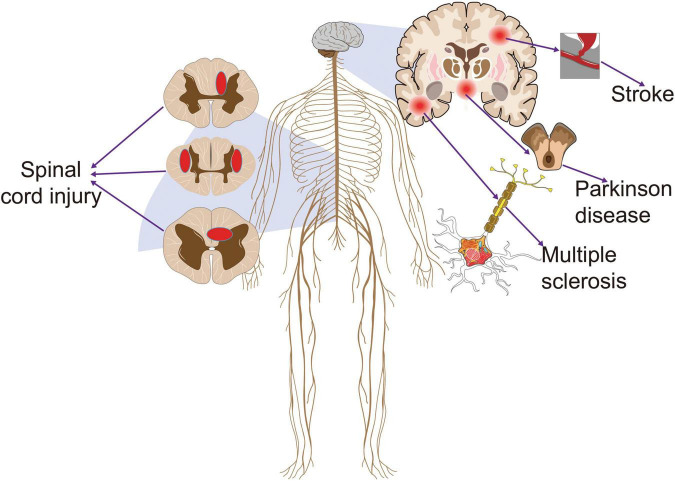
The common diseases that cause central neuropathic pain. Stroke, Parkinson’s disease, multiple sclerosis, and spinal cord injury often lead to central neuropathic pain that persists throughout the recovery cycle. This pain clearly has a negative effect on the prognosis of patients.

Neuropathic pain (NP) was defined by the International Association for the Study of Pain (IASP) in 2008 as “pain caused by a lesion or disease of the somatosensory nervous system” (Beydoun, 2003). And the prevalence of NP was about 3.3%–8.2% (Haanpää et al., 2011). According to the anatomical location of the injury or disease, NP can be classified as central NP (CNP), which is due to lesions or diseases of the spinal cord or brain, and peripheral neuropathic pain (PNP), which includes diabetic neuropathy, nerve damage, facial pain, phantom limb pain, cancer pain, and deformity (Colloca et al., 2017). The most common CNP syndromes include NP associated with spinal cord injury (SCI), post-stroke pain (PSP), NP associated with multiple sclerosis (MS), and Parkinson’s disease (PD) (Finnerup et al., 2016; Zhang et al., 2021; Figure 1). The appearance and aggravation of pain symptoms often occur within a few days after the lesion or disease. CNP has no specific treatment at present (Dworkin et al., 2013; Cruccu et al., 2016), and patients suffer from chronic pain for a long time, which seriously affects their quality of life. The aversive experience of pain is activated by the temporal and spatial coordination of a neural network called the pain matrix after a nociceptive stimulus (Garcia-Larrea and Bastuji, 2018). Pain matrix is mainly distributed in the cerebral cortex and subcortical regions, including thalamus, insula, cingulate cortex, prefrontal cortex, and frontal-orbitofrontal cortex (Mouraux et al., 2011). Responses during NP exhibit reproducible patterns, in particular hypoactivity of the thalamus contralateral to the pain area and deficit in reactiveness of the prefrontal cortices during NP (Garcia-Larrea and Peyron, 2013). Common pharmacological treatments for NP include calcium channel modulators, opioid analgesics, and antidepressants, while non-pharmacological treatments include exercise, noninvasive brain stimulation (NIBS), spinal cord stimulation (SCS), radiofrequency ablation (RFA), and nerve block (Baron et al., 2010; Reimer et al., 2014; Zheng et al., 2021; Peng et al., 2022; Wu et al., 2022). Since there is no specific drug for NP at present, non-drug therapy has been gradually accepted by NP patients because of its no side effects, no drug resistance, and strong pertinence (Moisset et al., 2020). NIBS has been applied in the rehabilitation of various brain dysfunction to regulate cortical excitability and neuroplasticity and has attracted wide attention because of its noninvasiveness, tolerability, and portability (Chisari et al., 2014). In basic and clinical settings, two approaches have become the pillars of NIBS: repetitive transcranial magnetic stimulation (rTMS) and transcranial direct current stimulation (tDCS) with a painless current (current intensities ± 1–2 mA) applied to the scalp. Both techniques are effective in reducing pain as measured by the visual analog scale (VAS) and numerical rating scale (NRS) (Ayache et al., 2016b; Nardone et al., 2017). rTMS focuses on neuromodulation sequelae through magnetic fields (Hallett, 2007). tDCS is applied to the scalp by a weak current to produce neuromodulation (Terney et al., 2008). In terms of different types of pain, NP response to NIBS is better than non-neurotic pain (Knotkova et al., 2021). NIBS is a promising therapeutic technique for resolving the dynamic neurological changes caused by NP (Costa et al., 2019).

## Repetitive Transcranial Magnetic Stimulation for Central Neuropathic Pain

Based on the principles of electromagnetic induction and electromagnetic conversion, TMS, including single-pulse TMS and rTMS, alters the motor potential of cortical nerve cells by stimulating the magnetic field generated by coil transients, which affects intra-brain metabolism and neuroelectric activity (Hallett, 2007). Compared with the chronic implantation procedure, rTMS is a safe, noninvasive, easy to tolerate, and effective therapeutic intervention pattern that continuously distributes multiple pulses at a fixed frequency and is more widely used in clinical applications (Gu and Chang, 2017; Choi et al., 2018). The main limitation of rTMS is the short-term analgesic effects (Hemond and Fregni, 2007). Low-frequency rTMS (LF-rTMS, ≤1 Hz) can inhibit the metabolism of nerve cells and reduce cortical excitability, whereas high-frequency rTMS (HF-rTMS, ≥1 Hz) has the opposite effect (Wagner et al., 2007). The reduction in VAS and NRS scores with HF-rTMS (10–20 Hz) is much greater than that with LF-rTMS (≤1 Hz) under conditions for analgesia (Lefaucheur et al., 2001; Canavero et al., 2002; Lefaucheur, 2006; Leo and Latif, 2007; Borckardt et al., 2011). Therefore, HF-rTMS is often used in the clinical treatment of NP. More research information about the effect of rTMS on CNP is shown in Table 1.

**TABLE 1:** Major findings of Repetitive transcranial magnetic stimulation (rTMS) in central neuropathic pain (CNP) studies.

Author, year	Study type	CNP type	Sample (size, sex, age)	rTMS site	Frequency/Intensity	Duration	Analgesic effect
de Oliveira et al., 2014	Prospective cohort	CPSP	21, 10M, 11F, Real: 55 ± 9.67, Sham: 57.8 ± 11.86	M1/DLPFC	10 Hz/120%RMT	10 days	No effective pain relief by VAS
Zhao et al., 2021	Randomized control	Acute CPSP	38, 21M, 7F, Real: 50.1 ± 11.34, Sham: 48.9 ± 11.51	Upper limb area of the motor cortex	10 Hz/80%RMT	3 weeks	Significant pain relief by NRS and MPQ
Khedr et al., 2005	Randomized parallel	PSP	24, 14M, 10F, 52.3 ± 10.3	Hand area of the motor cortex	20 Hz/80%RMT	5 days	Pain relief by VAS and LANSS scales
Hosomi et al., 2013	Cross-over	PSP	57, Gender not, 60.7 ± 10.6	M1	5 Hz/90%RMT	10 sessions	Modest pain relief by VAS
Hasan et al., 2014	Case series	PSP	14, 10M, 4F, 57 (median)	M1	10 Hz/80%–90%RMT	5 sessions	Modest but significant pain relief by NRS
Kobayashi et al., 2015	Cross-over	PSP	18, 12M, 6F, 63.0 ± 9.9	M1	5 Hz/90%RMT	12 weeks	Pain relief by VAS
Matsumura et al., 2013	Cross-over	PSP	20, 12M, 8F, 63.6 ± 8.1	M1	5 Hz/100%RMT	1 day	Pain relief by VAS correlated well with morphine test
Galhardoni et al., 2019	Cross-over	PSP	98, Gender not, 55.02 ± 12.13	ACC/PSI	10 Hz/90%RMT	5 sessions	No significant pain relief by NRS
Ohn et al., 2012	Case series	PSP	22, 13M, 9F, 54.0 ± 9	M1	10 Hz/90%RMT	5 days	Significant pain relief by VAS
Sun et al., 2019	Sham-control	SCI	17, 15M, 2F, 23.0–54.5	M1	10 Hz/80%RMT	6 weeks	More pain relief from 2 to 6 weeks by NRS
Lefaucheur et al., 2004	Cross-over	SCI	12, Gender/age not	M1	10 Hz/80%RMT	1 session	Significant but transient pain relief by VAS
Defrin et al., 2007	Randomized control	SCI	11, 7M, 4F, 54.0 ± 6	vertex	5 Hz/115%RMT	10 days	Continued pain relief by MPQ
Kang et al., 2009	Cross-over	SCI	11, 6M, 5F, 54.8 ± 13.7	M1	10 Hz/85%RMT	5 days	No Significant pain relief by NRS and BPI
Jetté et al., 2013	Cross-over	SCI	16, 11M, 6F, 50.0 ± 9	motor cortex (hand / leg area)	10 Hz/90%RMT (hand area) 110%RMT (leg area)	3 sessions	Significant but equivalent pain relief by NRS
Yılmaz et al., 2014	Randomized control	SCI	16, 16M, 38.6 ± 6.5	motor cortex	10 Hz/110%RMT	10 days	Middle-term (over 6 weeks) pain relief by VAS
Centonze et al., 2007	Randomized control	MS	19, 5M, 14F, Age not	M1	5 Hz/100%RMT	2 weeks	Long-lasting spasticity pain relief

*CNP, central neuropathic pain; PSP, post-stroke pain; CPSP, central post-stroke pain; SCI, spinal cord injury; MS, multiple sclerosis; M, male; F, female; rTMS, repetitive transcranial magnetic stimulation; Hz, hertz; RMT, resting motor threshold; ACC, anterior cingulate cortex; PSI, posterior superior insula; M1, primary motor cortex; DLPFC, dorsolateral prefrontal cortex; PMC, premotor cortex; VAS, visual analog scale; NRS, numerical rating scale; BPI, brief pain inventory; MPQ, McGill pain questionnaire; LANSS, Leeds assessment of neuropathic symptoms and signs.*

The guidelines of the International Federation of Clinical Neurophysiology and the European Federation of Neurological Societies supported the specific analgesic effect of HF-rTMS stimulation in the primary motor cortex (M1) of NP (Cruccu et al., 2007; Lefaucheur et al., 2020). Moreover, a longer course of treatment and continuous treatment was more conducive to the analgesic effect and therefore included in the grade A recommendation. Stimulation of the frontal lobes, particularly the dorsolateral prefrontal cortex (DLPFC), was associated with improved depression and cognitive impairment, but its effect on NP improvement was clinically controversial and not addressed in the guidelines. In the studies included in the guidelines, rTMS was often used to stimulate the contralateral M1 of NP at a frequency of 5–10 Hz; approximately 80%–90% resting motor threshold (RMT) and 5–10 sessions of treatment can usually have a definite analgesic effect (Lefaucheur et al., 2020).

### Repetitive Transcranial Magnetic Stimulation for Post-stroke Pain

Pain was common and present in 10%–45.8% of stroke cases (Paolucci et al., 2016; Choi-Kwon et al., 2017). PSP impeded recovery, affects the mental state of patients with stroke, and further impairs the quality of life of patients (Lundström et al., 2009; Naess et al., 2012). The common variants of PSP are central poststroke pain (CPSP), complex regional pain syndrome (CRPS), shoulder pain, spasticity-related pain, and headache (Treister et al., 2017; Delpont et al., 2018). CPSP was a CNP disorder that affected 10%–35% of the post-stroke population (Flaster et al., 2013). The properties of pain included searing or freezing pain or numbness, and pain intensity was reported as a VAS score of nearly 8 out of 10 (Oh et al., 2015). Some studies that focused on the analgesic effect of rTMS on PSP showed that HF- rTMS (5–20 Hz) can produce effective immediate pain relief in patients after stroke, and multiple sessions and a long duration of intervention can make the analgesic effects last (Ohn et al., 2012; Hosomi et al., 2013; Matsumura et al., 2013; Hasan et al., 2014; Kobayashi et al., 2015; Ramger et al., 2019). Most patients with CPSP responded positively to rTMS (Ohn et al., 2012; Matsumura et al., 2013; Kobayashi et al., 2015). Pain relief was more pronounced in the rTMS group than in the sham stimulation group as measured by VAS and NRS. They also found a time-course effect on pain relief after 1 and 3 weeks of stimulation that lasted for up to 12 weeks and peaked at around 8 weeks.

The influence of the site of the stimuli around the motor cortex on the analgesic effect is also the focus of research (Saitoh and Yoshimine, 2007). M1 is the stimulus area selected by most studies and has achieved a good analgesic effect on PSP (Saitoh and Yoshimine, 2007; Ohn et al., 2012; Hosomi et al., 2013; Matsumura et al., 2013; Hasan et al., 2014; Sacco et al., 2014; Kobayashi et al., 2015; Ramger et al., 2019). In addition, two studies (Khedr et al., 2005; Zhao et al., 2021) selected the upper limb and hand regions of the motor cortex as stimulation sites and found that CPSP is effectively alleviated by intervention at these sites. By contrast, DLPFC and anterior cingulate cortex (ACC) were also selected for stimulation in some studies but did not produce analgesic effects compared with the stimulation of the motor cortex (de Oliveira et al., 2014; Galhardoni et al., 2019; Attal et al., 2021). In addition to common PSP, Choi and Chang (2018) found that rTMS could be used as an effective therapeutic tool for managing post-stroke shoulder pain, and this pain relief could be maintained for about 4 weeks after 10 sessions of HF-rTMS (10 Hz) treatment.

### Repetitive Transcranial Magnetic Stimulation for Central Neuropathic Pain Associated With Spinal Cord Injury

Central neuropathic pain is a common and disabling symptom in individuals with SCI; it affects 75%–81% of SCI patients, and one-third reported severe pain that worsens their mood state (Margot-Duclot et al., 2009). CNP affects the quality of life, rehabilitation, and recovery of more than two-thirds of SCI cases (Rekand et al., 2012). CNP following SCI is resistant to common pharmacologic treatments (Moreno-Duarte et al., 2014). rTMS has been developed to offer a safe and reliable approach to pain management (Quesada et al., 2018). Most of the stimulation sites were located in M1, as well as the premotor cortex (PMC) and limb cortex areas depending on the pain site (Lefaucheur et al., 2004; Sun et al., 2019). One study found analgesic effects when stimulating the vertex (Defrin et al., 2007). HF-rTMS (5–20 Hz) can produce effective pain relief for NP following SCI (Kang et al., 2009; Yılmaz et al., 2014; Zhao et al., 2020). Zhao et al. (2020) found that 10 Hz rTMS over the hand area of the motor cortex could relieve acute CNP during the early stage of SCI. Some studies indicated that rTMS has no early pain relief after SCI but has a better intermediate analgesic effect compared with sham rTMS (Shen et al., 2020). Regarding the management of intractable NP by rTMS in cases with SCI, Yılmaz et al. (2014) found that the middle-term (over 6 weeks) analgesic effect of rTMS (10 Hz) was encouraging. Sun et al. (2019) found that rTMS (10 Hz, 6 weeks with 1-day interval per week) showed more analgesic effect on NP following SCI at 2–6 weeks. rTMS applied over the hand or leg motor cortex can relieve NP, improve spasm, and therefore reduce pain in patients with incomplete SCI (Kumru et al., 2010; Jetté et al., 2013). Pain relief caused by single rTMS treatment may be due to placebo, but patients with SCI may benefit from multiple rTMS sessions (Defrin et al., 2007).

### Repetitive Transcranial Magnetic Stimulation for Central Neuropathic Pain Associated With Parkinson’s Disease

Parkinson’s disease is a common chronic progressive neurodegenerative disease among middle-aged and elderly people and is characterized by motor and non-motor symptoms. More than 90% of patients with PD experienced non-motor symptoms; among which, the most common was pain with an incidence of 40%–85%, and it seriously affected the quality of life of patients (Elefant et al., 2012; Young Blood et al., 2016). The current rTMS protocol did not pose a substantial risk to patients with PD (Vonloh et al., 2013). Most current research on rTMS in PD focused on motor symptoms, such as gait, motor retardation, and coordination, as well as emotional and psychiatric symptoms (Xie et al., 2015; Brys et al., 2016; Dagan et al., 2017). rTMS has antidepressant efficacy and can improve motor function (Xie et al., 2015). Studies showed that PD with NP was associated with depression and dyskinesia; thus, treating depression to improve motor symptoms can relieve the pain of patients with PD (Yang et al., 2014). rTMS usually targets the left DLPFC in the treatment of depression but regulates the excitability of pain circuits in related brain regions by stimulating the M1 region to achieve analgesia (Moseley and Flor, 2012; Martin et al., 2013). Therefore, the study of the analgesic effect of rTMS on patients with PD still needs more research input in terms of stimulation site, as well as the frequency and intensity of stimulation.

### Repetitive Transcranial Magnetic Stimulation for Central Neuropathic Pain Associated With Multiple Sclerosis

Multiple sclerosis is a disease caused by an inflammatory demyelinating process in the central nervous system (CNS) and a leading cause of disability in young adults with substantial economic and social burdens (Patwardhan et al., 2005; Yamout and Alroughani, 2018). Many common symptoms of MS, such as spasticity, pain, depression, and cognitive impairment, cannot be fully managed by medication (Kesselring and Beer, 2005). The first clinical application of rTMS in MS patients was to manage spasms (Centonze et al., 2007). HF-rTMS can significantly reduce spasticity, compared to sham stimulation. Some studies supported a more durable effect in reducing pain and fatigue following HF-rTMS (Mori et al., 2011; Korzhova et al., 2019). According to the recent evidence-based guidelines (Lefaucheur et al., 2014), no recommendations still exist for the use of rTMS in the treatment of patients with MS, even though rTMS has few promising results for sensory and motor symptoms (Iodice et al., 2017).

### Mechanisms of Repetitive Transcranial Magnetic Stimulation for Central Neuropathic Pain

**FIGURE 2:**
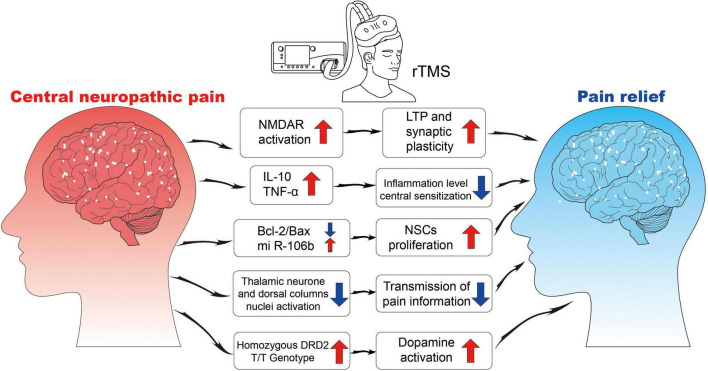
The neurophysiological mechanisms of rTMS. NMDA, N-methyl-D-aspartate receptor; LTP, long-term potentiation; IL, interleukin; TNF, tumor necrosis factor; Bcl, B-cell lymphoma; Bax, Bcl2-associated X; NSCs, neural stem cells; DRD2, dopamine receptor D2.

Currently, the mechanism of rTMS is found to be related to synaptic plasticity, neural regulation, and response (Figure 2).

#### Brain Plasticity

Repetitive transcranial magnetic stimulation acts on the synapses of neurons and causes axons to be depolarized and transmitted down-line, which then leads to changes in the nerve cell body, cell permeability, and excitability and results in brain plasticity. Esser et al. (2006) found that HF-rTMS induces long-term potentiation (LTP), which is a change in information transmission resulting from the emergence of central neurologic transsynaptic/trans-presynaptic nerve fibers and is also one of the important cellular and molecular mechanisms of human memory and learning. HF-rTMS can remarkably increase the level of serum brain-derived neurotrophic factor (BDNF) by enhancing cortical excitability and brain plasticity, which may be the basis of NP treatment by HF-rTMS (Dockx et al., 2018). Blocking signaling between BDNF and Tyrosine receptor kinase B (TrkB) was found to reduce abnormal pain caused by nerve injury (Coull et al., 2005). But rTMS has been found to enhance this signaling in the cerebral cortex (Wang et al., 2011). rTMS may induce the increase of BDNF level in the motor cortex or frontal lobe to cause neuroplasticity changes and thus achieve the analgesic effect.

Ghosh et al. (2010) found that rTMS may regulate the balance of inhibitory neurotransmitters and excitatory glutamate neurotransmitters in the cerebral cortex to achieve pain relief. Gamma-aminobutyric acid (GABA) is an inhibitory neurotransmitter because it inhibits certain interneuronal synaptic signals, thereby preventing or reducing the risk of CNP (Gwak and Hulsebosch, 2011). Some studies found that single rTMS protocol increased phrenic motoneuron excitability at 10 Hz through the mediation of a local GABA ergic disinhibition (Barr et al., 2013; Michel-Flutot et al., 2021). rTMS (10 Hz) alleviated acute CNP in the early stages of SCI by improving motor-evoked potential (MEP) parameters and modulating BDNF and nerve growth factor (NGF) secretion (Zhao et al., 2020). Some studies have found that rTMS can promote dopamine release and dopamine activity is affected by the DRD2 genotype (Strafella et al., 2001, 2003; Hagelberg et al., 2002). When navigated rTMS targeted M1, the participants with homozygous DRD2 T/T genotype were remarkably more likely to experience pain relief than those with other genotypes (Ojala et al., 2021). Therefore, the plasticity-related gene polymorphisms of DRD2 may play a key role in CNP regulation.

#### Central Sensitization Reduction

Central sensitization refers to the abnormal increase in the excitability or synaptic transmission of central pain-related neurons, including the increase in the spontaneous discharge activity of neurons, the expansion of the sensory domain, the reduction of threshold value to external stimuli, and the enhancement of response to suprathreshold stimuli, which amplify the transmission of pain signals (Dooley et al., 2007; Latremoliere and Woolf, 2009; Quintero et al., 2011; Nickel et al., 2012). The maintenance of NP depends on central sensitization. Cioni and Meglio (2007) used functional magnetic resonance imaging to find that rTMS can inhibit the transmission of pain information in the spinothalamic pathway. HF-rTMS may reduce central sensitization and relieve NP by down-regulating the overexpression of neuronal nitric oxide synthase in ipsilateral dorsal root ganglions and inhibiting the activity and proliferation of astrocytes in L4–6 spinal dorsal horn ipsilateral to the NP (Yang et al., 2018).

#### Neuroinflammation Modulation

The exudation of mast cells, macrophages, and other immune cells; sympathetic nerve excitation; and vascular dilation after nerve injury or disease make the peripheral nervous system and CNS produce histamine, NGF, IL-10, tumor necrosis factor (TNF)-α, and other pro-inflammatory cytokines and then cause NP (Vallejo et al., 2010; Li et al., 2011). Mechanical ectopic pain and hyperalgesia were partially reversed by rTMS, which may be related to increased levels of TNF-α, and IL-10 in the prefrontal cortex (Toledo et al., 2021).

Tumor necrosis factor-α expression in the central nervous system contributes to the induction of NP in rats (Andrade et al., 2011). The classical anti-inflammatory effects of IL-10 may be involved in the development of NP (Moore et al., 2001). rTMS has been shown to alter IL-10 levels in a variety of situations. rTMS reduced the activation of microglia and increased the level of IL-10 in the cortex, alleviating neurological abnormalities in rats with MS-induced neurological injury (Yang et al., 2020). The reduction of neurotoxic astrocyte polarization through IL-10 effects has been proposed as a mechanism by which rTMS (5-10 Hz) is effective in nerve regeneration induced by stroke in rats (Hong et al., 2020). Changes in IL-10 levels observed in NP rats treated with rTMS are reflected in increased TNF-α, contributing to central homeostasis. A link between pain and microglia TNF-α has been proposed because it improves long-term synaptic enhancement in spinal horn C fibers in animal models of nerve injury (Liu et al., 2017).

#### Cell Proliferation

Repetitive transcranial magnetic stimulation could promote nerve cell proliferation in healthy, depressed, and stroke rat models. Ueyama et al. (2011) stimulated rats with HF-rTMS (25 Hz) for 2 weeks and found that 5-bromo-2-deoxyuridine-positive cells in the subventricular zone increased remarkably in the rTMS group. The proliferative cells were later identified as neural stem cells (NSCs), but the proliferative mechanism remains unclear. The anti-apoptotic effect of rTMS may cause NSC proliferation. Yoon et al. (2011) treated cerebral ischemia rats with HF-rTMS (10 Hz) for 14 days, and the Bcl-2/Bax ratio increased and apoptosis decreased after treatments. In addition, Guo et al. (2014) found that 10 Hz rTMS can promote the secretion of the miR-106B family in cerebral ischemia rats and regulate the NSC cycle by regulating the downstream target gene, P57, which indicates that HF-rTMS can affect the cell cycle and stimulate cell proliferation. The in vitro stimulation of NSCs by HF-rTMS (10 Hz) can increase the mIR-106B expression of NSCs and promote the proliferation of NSCs (Liu et al., 2015). However, no relevant experimental study has been conducted on whether rTMS can promote NSC differentiation.

## Transcranial Direct Current Stimulation for Central Neuropathic Pain

Transcranial direct current stimulation is an approach that induces neuroplasticity and modulates cortical excitability by applying a weak direct current over the scalp of subjects (Stagg and Nitsche, 2011). tDCS is a noninvasive neuromodulatory technique that reduces bidirectional polarity-dependent changes in underlying cortical areas (Fregni et al., 2006). Five studies, which collectively included 95 NP cases, also found that tDCS can effectively manage NP (Antal et al., 2010; Soler et al., 2010; Bolognini et al., 2013; Attal et al., 2016; Houde et al., 2020). In the included studies, the M1 area plays a key role in the analgesic effect of tDCS. tDCS’s cathode stimulation of the M1 and PMC can improve the hand motor function of patients with stroke, as well as the pain perception and pain-related symptoms induced by chronic pain (Andrade et al., 2017; Zortea et al., 2019). tDCS is a safe technique and has slight adverse reactions, such as headache, discomfort on the scalp, and a slight burning sensation under the electrode sheet (Nitsche et al., 2009; Brunoni et al., 2011; Fagerlund et al., 2015). Compared with rTMS, tDCS does not have the risk of convulsions and only has a brief and mild tingling sensation, whereas rTMS causes tingling throughout the process (Hummel et al., 2005).

The tDCS guidelines published by the International Society for Neuropsychopharmacology indicated that the use of tDCS to stimulate the left M1 region was highly effective in improving NP and was therefore a level B recommendation (Fregni et al., 2021). The guideline of the International Federation of Clinical Neurophysiology pointed out that tDCS in M1 (contralateral to pain side) in chronic lower limb NP following SCI was a level C recommendation (possible efficacy) (Lefaucheur et al., 2017). The commonly used tDCS parameter was an intensity of 2 mA, which was performed continuously for at least 5 consecutive days with a duration of 20 min each time. Although the current level of evidence suggests that tDCS was less effective than rTMS in relieving pain when stimulating the M1 area, the most surprising point was that tDCS appeared to be more effective for NP following SCI in the lower extremities (Lefaucheur et al., 2004, 2006, 2014, 2017). This point was reinforced by the treatment of a patient with chronic refractory NP who did not respond to the HF-rTMS but gradually improved by the tDCS over a long period (Hodaj et al., 2016). More research information about the effect of tDCS on CNP is shown in Table 2.

**TABLE 2:** Major findings of transcranial direct current stimulation (tDCS) in central neuropathic pain (CNP) studies.

Author, Year	Study type	CNP type	Sample (size, sex, age)	tDCS site	Intensity/Current flow time	Duration	Analgesic effect
Bae et al., 2014	Randomized control	PSP	14, 7M, 7F, 45–55	M1	2 mA/20 min	3 weeks	Pain relief by VAS
Hassan et al., 2021	Case report	PSP	1, Gender not, 45	DLPFC	2 mA/20 min	2 weeks	Immediate but transient pain relief by VAS
Molero-Chamizo et al., 2021	3 cases report	PSP	3, 2W:43, 72, 1M:54	M1	1.5 mA/20 min	5 sessions	Significant pain relief by AVAS
Fregni et al., 2006	Randomized control	SCI	17, 14M, 3F, 35.7 ± 13.3	M1	2 mA/20 min	16 days	Significant pain relief by VAS, CGI and PGA
Soler et al., 2010	Randomized control	SCI	39, 30M, 9F, 44.1 ± 11.6	M1	2 mA/20 min	10 sessions	Effective relief for continuous and paroxysmal pain by NRS and BPI
Wrigley et al., 2013	Cross-over	SCI	10, 8M, 2F, 56.1 ± 14.9	M1	2 mA/20 min	5 days	Effective relief for recent pain by NRS
Yoon et al., 2014	Prospective control	SCI	16, 12M, 4F, 44.1 ± 8.6	M1	2 mA/20 min	10 days	Significant pain relief by NRS
Ngernyam et al., 2015	Cross-over	SCI	20, 15M, 5F, 44.5 ± 9.16	M1	2 mA/20 min	1 session	Significant pain relief by NRS
Thibaut et al., 2017	Two-phase randomized control	SCI	Phase I: 33, 24M, 9F, 51.2 ± 12.5 Phase II: 9, 7M, 2F, 49.0 ± 14.38	M1	2 mA/20 min	5 days (Phase I) 10 days (Phase II)	Long-lasting pain relief by VAS
Mori et al., 2010	Randomized, sham-control	MS	19, 8M, 11F, 23–68	motor cortex	2 mA/20 min	5 days	Significant pain relief by VAS and MPQ
Ayache et al., 2016a	Cross-over	MS	16, 3M, 13F, 48.9 ± 10.0	DLPFC	2 mA/20 min	3 days	Significant pain relief by VAS and BPI
González-Zamorano et al., 2021	Randomized control	PD	32, Gender not, Age not	M1	2 mA/20 min	2 weeks (10 sessions)	Pain relief by BPI, KPDPS, PPT, TS and CPM

*CNP, central neuropathic pain; PSP, post-stroke pain; CPSP, central post-stroke pain; SCI, spinal cord injury; MS, multiple sclerosis; PD, Parkinson’s disease; M, male; F, female; tDCS, transcranial direct current stimulation; RMT, resting motor threshold; mA, milliampere; min, minute; M1, primary motor cortex; DLPFC, dorsolateral prefrontal cortex; VAS, visual analog scale; AVAS, adaptive visual analog scale; NRS, numerical rating scale; BPI, brief pain inventory; MPQ, McGill pain questionnaire; CGI, clinical global impression; PGA, patient global assessment; PPT, pain pressure threshold; KPDPS, King’s Parkinson’s disease pain scale; TS, temporal summation; CPM, conditioned pain modulation.*

### Transcranial Direct Current Stimulation for Post-stroke Pain

Patients with CPSP may have skin temperature changes because of autonomic nervous system dysfunction. Therefore, the physiological changes in patients with CPSP can be evaluated by measuring the skin temperature difference between the pain area and contralateral area (Klit et al., 2009; Kim, 2014). Bae et al. (2014) found a 1.15 reduction in pain intensity through VAS after 3 weeks of treatment in the tDCS group, as well as changes in warm sensation and cold pain threshold, some of which lasted up to 3 weeks after stimulation. This result means that tDCS improved sensory identification and exerted analgesic effects in patients with stroke and PSP. The decreased skin temperature also reduces the sensitivity of patients to pain and thus contributes to analgesia (Tan and Knight, 2018; Madden and Morrison, 2019). Ramger et al. (2019) also suggested that tDCS on M1 has positive effects on CPSP.

### Transcranial Direct Current Stimulation for Central Neuropathic Pain Associated With Spinal Cord Injury

The increased excitability and reactivity of spinal dorsal horn neurons caused by the dysregulation of the central inhibitory mechanism is an important cause of pain following SCI (Tung et al., 2015). Fregni et al. (2006) first reported the effect of tDCS on NP after SCI. Subjects were randomly divided into the tDCS group (2 mA, 20 min) and the sham stimulation group to receive motor cortex stimulation. After 5 days of stimulation, the pain was remarkably reduced in the tDCS group according to the VAS, whereas no considerable change was observed in the sham stimulation group. More recently, Murray et al. (2015) investigated the effect of different current intensities on tDCS in treating NP after SCI. The subjects were randomly assigned to different groups (1 mA, 2 mA, and sham stimulation of the motor cortex). They found that MEP increased considerably in minutes only after 2 mA tDCS motor cortex stimulation. From this result, we can speculate that current intensity may influence the clinical outcome of tDCS stimulation. In addition, Soler et al. (2010) applied visual illusion technology and tDCS to patients with CNP after SCI, and the combination of the two can relieve pain more effectively than monotherapy.

Some evidence showed that tDCS did not provide any pain relief to longstanding NP after SCI (Wrigley et al., 2013). The analgesic effect of tDCS was not superior to exercise alone after 12 sessions of intervention, and the beneficial effect was not maintained at follow-up (Mehta et al., 2015; Yeh et al., 2021). However, not enough evidence could suggest that the analgesic effect of tDCS on NP following SCI over the M1 region is effective compared with medication treatments because of the lack of high-quality studies and sufficient sample size and control groups (Boldt et al., 2014; Nardone et al., 2014; Mehta et al., 2015; Ngernyam et al., 2015; David et al., 2018; Shen et al., 2020; Li et al., 2021). Fagerlund et al. (2015) found that pain reduction after the tDCS stimulation of the motor cortex was closely associated with increased peak spectral density in the θ-α range of electroencephalogram, but no corresponding association was found with sham stimulation. This finding may become a measurement tool to quantify the effect of tDCS management on NP to better compare analgesic effects.

### Transcranial Direct Current Stimulation for Central Neuropathic Pain Associated With Parkinson’s Disease

Transcranial direct current stimulation manages PD through brain stimulation by very weak currents to activate neurons into an excitable state (Shigematsu et al., 2013). A growing number of studies have shown that tDCS can improve motor and cognitive symptoms, but the results suggest that a fully optimized tDCS protocol has not been established (Biundo et al., 2016; Elsner et al., 2016; Putzolu et al., 2018; Broeder et al., 2019; Orru et al., 2019). Few studies have been conducted on tDCS for PD-related pain, but home-isolated patients with PD are experiencing increased pain frequency because of the reduction of movement due to the protocols for the coronavirus 2019 (COVID-19) pandemic. Approximately 49.7% of Spanish patients with PD reported pain every day during the COVID-19 pandemic (Santos-García et al., 2020). González-Zamorano et al. (2021) proposed a new method based on pain psychological expression techniques and tDCS in patients with pain following PD. Finally, after the configuration and explanation, the treatment can be applied at home to promote independence and self-management and maximize the time out of medical centers.

### Transcranial Direct Current Stimulation for Central Neuropathic Pain Associated With Multiple Sclerosis

Transcranial direct current stimulation has been gradually used in the clinical of spasticity and pain in MS (Mori et al., 2010; Dubbioso et al., 2015; Iodice et al., 2015; Rossini et al., 2015; Saturnino et al., 2015; Ayache et al., 2016b). Poorly managed spasticity can lead to pain and limited mobility. Mori et al. (2010) researched the effect of the application of tDCS (2 mA, 20 min/day, 5 sessions) over M1 contralateral to the affected side on chronic, drug-fast pain. Nineteen patients with MS were randomized to receive sham stimulation or tDCS. Remarkable pain relief was found following tDCS but not sham stimulation as measured by VAS and McGill questionnaire, and a total improvement in quality of life was observed within 3 weeks after the end of treatment. tDCS could have acted on the pain matrix networks where the prefrontal cortex mainly contributed (Ayache et al., 2016b). And anodal tDCS over the DLPFC appeared to increase the pain threshold to produce analgesic effects, especially NP. Overall, data for the treatment of NP following MS with tDCS is sparse (Palm et al., 2014).

### Mechanisms of Transcranial Direct Current Stimulation for Neuropathic Pain

**FIGURE 3:**
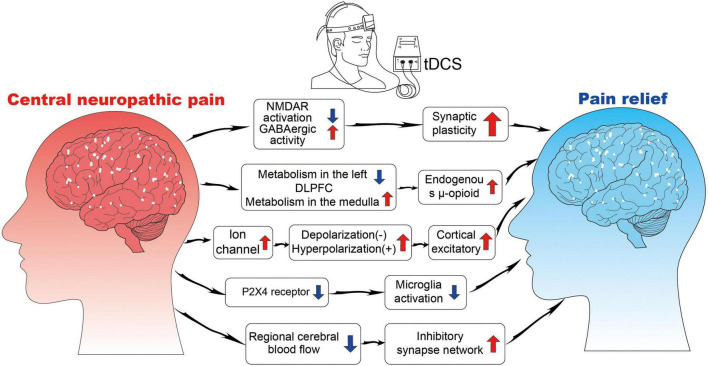
The neurophysiological mechanisms of tDCS. NMDAR, N-methyl-D-aspartate receptor; GABA, gamma-aminobutyric acid; P2X4, purinoceptor 4.

The effect of tDCS in reducing CNP may be related to increased sympathetic nerve activity, decreased blood flow, and decreased or interrupted transmission of the connection between sympathetic nerve fibers and pain-transmitting nerve fibers (Figure 3).

#### Selective Excitability of Neurons

Transcranial direct current stimulation is thought to work by changing the excitability of nerve cells as electricity passes through brain tissue (Nitsche et al., 2008; To et al., 2016; Stagg et al., 2018). The current intensity used by tDCS is weak, does not cause action potential, only changes the resting membrane potential of nerve cells, and regulates the excitability of nerve cells (Bikson et al., 2004). tDCS affects the opening and closing of ion channels in the stimulated region and induces the flow of intracranial ions. Anodic stimulation leads to the depolarization of the nerve membrane, whereas cathodic stimulation leads to the hyperpolarization of the nerve membrane, both of which change the excitability of neurons (Zaghi et al., 2010). This effect can occur a few seconds after tDCS stimulation; therefore, it is often referred to as the immediate effect of tDCS stimulation (Chang et al., 2015). Notably, the cortical excitatory effect of tDCS is related to the direction and intensity of the stimulus current, but the relationship is not linear, that is, a greater current intensity has a better stimulus effect, but sometimes, the effect will be reversed with the increase in current intensity (Batsikadze et al., 2013; Benwell et al., 2015). The excitatory effect of tDCS on neurons is selective to some extent, that is, tDCS only acts on neurons that are already in an active state. This feature of tDCS can effectively avoid the side effects of excitatory toxicity caused by traditional nerve stimulation techniques (Fertonani and Miniussi, 2017).

#### Synaptic Plasticity and Connectivity

The subsequent effects of the cessation of tDCS stimulation may be related to the regulation of synaptic plasticity and connect ability by regulating neurotransmitter activity (Nitsche et al., 2008; To et al., 2016). Synaptic plasticity involves glutamate and GABAergic neurons, which produce glutamate and GABA, respectively. Glutamate N-methyl-D-aspartate (NMDA) receptor agonist, D-cycloserine, prolongs the effect of tDCS on M1 excitability (Nitsche et al., 2004a). GABA receptor agonist, lorazepam, enhances and extends the subsequent effects of tDCS in a short period (Nitsche et al., 2004b). tDCS can induce long-term enhancement or inhibition in the stimulated region, which leads to synaptic remodeling, by regulating NMDA expression and GABA release (Antonenko et al., 2017). tDCS also has a network effect that can alter the structure and functional connections between different brain regions. Lin et al. (2017) found that the analgesic effect of tDCS is associated with the structural connections between the left DLPFC and the left thalamus. Cummiford et al. (2016) found that applying anode stimulation to the left M1 reduces the functional connection of the left abdominal extrinsic thalamus to the inner frontal lobe and the left auxiliary movement area, as well as the functional connection between the right abdominal extrinsic thalamus and the lower chin and the left auxiliary movement area, which play an important role in pain processing and regulation.

#### Regulation of Pain Receptor Expression

The activation of purinoceptor 4 (P2X4) receptors in the microglia is a sufficient and necessary condition for NP (Wasserman and Koeberle, 2009; Gritsch et al., 2016). The P2X4 receptor is expressed in the spinal cord ganglion and brain microglia, and its upregulation is the key process for the microglia to participate in NP (Baroja-Mazo et al., 2013). Microglia in the posterior horn of the ipsilateral spinal cord is activated rapidly, the expression of the P2X4 receptor is upregulated, and the change in P2X4 receptor expression is consistent with the timeline of mechanical pain sensitivity (Nasu-Tada et al., 2006; Beggs et al., 2012). tDCS can improve NP while inhibiting neuronal sensitivity and microglial activity after peripheral nerve injury (Zhang et al., 2020). This outcome may be due to the downregulation of P2X4 receptor expression by tDCS, which in turn inhibits microglia activity. Therefore, P2X4 can be used as a therapeutic target to treat NP in future studies.

#### Changes in Brain Blood Flow and Metabolism

Transcranial direct current stimulation modulates the activities of brain regions directly under the stimulating electrode, as well as a network of brain regions that are functionally related to the stimulated area. Zheng et al. (2011) found that the effect of tDCS is related to changes in cerebral blood flow, and blood flow is remarkably reduced and continues for a period after cathodic stimulation; these effects may also be a key mechanism of tDCS’s therapeutic role. Yoon et al. (2014) found increased metabolism in the medulla and decreased metabolism in the left DLPFC after active tDCS stimulation compared with sham tDCS. In addition, an increase in metabolism after active tDCS was observed in the subgenual anterior cingulate cortex and insula. An instant increase in the endogenous μ-opioid release may occur during acute motor cortex neuromodulation with tDCS (DosSantos et al., 2012).

## Limitations and Recommendations

The relevant NIBS studies still have several shortcomings, which may be important causes of the inconsistency in research results and difficulties in clinical application. First, most studies have small sample sizes (typically less than 40 people per group). Studies with a limited sample size may lead to the poor stability of the results and the inability to reliably reveal the true analgesic effect of NIBS because of the subjective characteristics of pain scores and differences in pain sensitivity among individuals. Future research should gradually carry out large-scale, multicenter studies to test the stability and reliability of the analgesic effects of NIBS.

Second, the current research on the analgesic effect of brain stimulation is not sufficient and in-depth. The analgesic research of rTMS is limited to CPSP, and the attention to other pain types is insufficient. The parameters used in the study of tDCS are relatively single, and the effect of stimulus parameters on analgesia is not clear. The effect of stimulation parameters on the regulation of analgesic effect should be comprehensively investigated. Micro-neuron discharge and neurotransmitter release can be integrated, as well as macro-brain response signal and somatic nervous system signal changes, through cross-species studies.

Third, understanding analgesic mechanisms rely on comparisons with analgesic loops found in other analgesic areas in the past without substantial evidence. Additionally, the influence of the placebo effect cannot be excluded because some experimental designs did not set a placebo group and only examined the changes in pain indexes before and after stimulation. Scale measurement or behavioral experiments can be carried out in multi-experimental and placebo groups to reveal the analgesic circuits of NIBS. In addition, the current assessment of the analgesic effect of different combinations of technologies is insufficient, and the interaction between NIBS and analgesic drugs is less considered. Combining different approaches may enhance analgesic effects by considering differences in pain-avoidance mechanisms.

## Discussion and Conclusion

In this article, the analgesic effects of common NIBS techniques on CNP are described in detail, and their respective analgesic mechanisms are discussed. The stimulation parameters for rTMS to produce an analgesic effect are stimulation frequency of 5–10 Hz, RMT of 80–90%, 5–10 times, and current intensity of 2 mA, 20–30 mins a time, 5–10 times is the common parameter of tDCS. The most popular stimulation area of analgesia is the M1 region for rTMS and tDCS. In addition, DLPFC has also been used as a target for NP improvement for tDCS. Although tDCS stimulation of the DLPFC region has been found to reduce pain caused by MS, current guidelines do not mention improving NP by stimulating DLPFC. tDCS with different parameters acting on the DLPFC region to reduce NP induced by various diseases needs further study.

**FIGURE 4:**
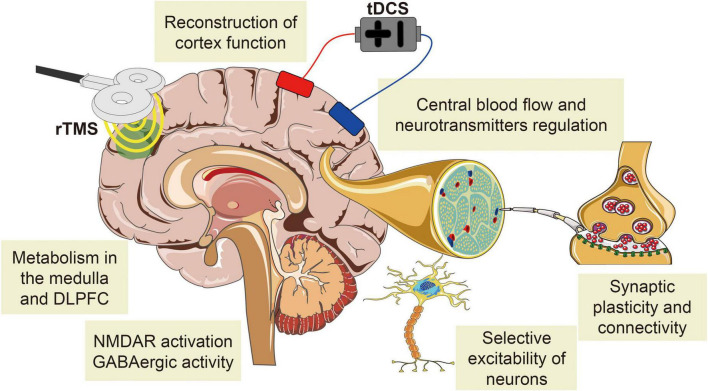
The common mechanisms of NIBS analgesia on CNP. The analgesic mechanism of rTMS is similar to that of tDCS, both of which change cortical excitability and synaptic plasticity, regulate the release of related neurotransmitters, and affect the structural and functional connections of brain regions associated with pain processing and regulation.

Repetitive transcranial magnetic stimulation analgesic research has been relatively mature and applied to a variety of CNP treatments (Lefaucheur et al., 2001; Lefaucheur, 2006; Ayache et al., 2016a; Nardone et al., 2017; Quesada et al., 2018; Sun et al., 2019). The analgesic mechanisms of rTMS and tDCS are similar, which both alter cortical excitability and synaptic plasticity, regulate the release of related neurotransmitters, and affect the structural and functional connections of brain regions associated with pain processing and regulation (Figure 4; Zheng et al., 2011; Thibaut et al., 2017; Hassan et al., 2021; Molero-Chamizo et al., 2021). tDCS has been studied in several studies and was able to manage pain effectively, but its optimal stimulation targets, stimulation intensity, and stimulation time for each type of CNP are still difficult to identify (Iodice et al., 2017; Wen et al., 2017; Young et al., 2020). NIBS not only affects the cerebral cortex at the stimulated site but also affects the related functional areas of the brain based on the pain matrix. The selection of optimal stimulation sites and parameters depends on the role of the primary disease-causing NP and its associated homologous brain regions in pain reduction. Revealing the interaction between NIBS with different parameters and cerebral cortex has great practical value for the selection of clinical analgesic methods to ultimately relieve pain and reduce the health and economic burden of pain on patients, families, and society.

## Author Contributions

X-QW and YF conceived the review. Q-HY drafted the manuscript and searched the literature to identify eligible trials. S-HD and Y-CW extracted and analyzed the data. Y-HZ revised the tables in the drafted manuscript. All authors approved the final manuscript.

## Conflict of Interest

The authors declare that the research was conducted in the absence of any commercial or financial relationships that could be construed as a potential conflict of interest.

## Publisher’s Note

All claims expressed in this article are solely those of the authors and do not necessarily represent those of their affiliated organizations, or those of the publisher, the editors and the reviewers. Any product that may be evaluated in this article, or claim that may be made by its manufacturer, is not guaranteed or endorsed by the publisher.
